# The association between the timing, intensity and magnitude of adolescent growth and body composition in early adulthood

**DOI:** 10.1038/s41430-023-01293-9

**Published:** 2023-06-13

**Authors:** Lukhanyo H. Nyati, John M. Pettifor, Ken K. Ong, Shane A. Norris

**Affiliations:** 1 SAMRC/Wits Developmental Pathways for Health Research Unit, Department of Paediatrics, Faculty of Health Sciences, University of the Witwatersrand, Johannesburg, South Africa; 2 Interprofessional Education Unit, Faculty of Community and Health Sciences, University of the Western Cape, Cape Town, South Africa; 3 MRC Epidemiology Unit and Department of Paediatrics, University of Cambridge School of Clinical Medicine, Box 285 Institute of Metabolic Science, Cambridge Biomedical Campus, Cambridge, CB2 0QQ UK; 4 School of Human Development and Health, University of Southampton, Southampton, UK

**Keywords:** Epidemiology, Obesity

## Abstract

**Objectives:**

There’s paucity of longitudinal studies assessing the role of adolescent growth on adult body composition in developing countries. The aims of this study were to assess the association between adolescent change in height, weight and BMI and early adult height, weight, body fat and lean mass.

**Methods:**

Magnitude, timing and intensity of height, weight and BMI growth were modelled for participants from the Birth to Thirty (Bt30) cohort (7–23 years). Early adult height, weight, BMI and DXA-derived body composition were obtained 1881 black participants (21–24 years). Linear regression analyses were used to assess associations.

**Results:**

Adolescents with an earlier onset of puberty were heavier in childhood and had an earlier timing and faster weight gain velocity in late adolescence. The intensity of adolescent weight gain was positively associated with adult BMI and fat mass index (FMI) in females. Early timing of adolescent BMI gain was associated with increased weight and BMI in adult females and FMI in adult males. Achieving peak weight velocity around age at peak height velocity was associated with lower BMI and fat mass in both sexes.

**Conclusion:**

This study confirms the adverse consequences of excessive weight gain prior to puberty, which is associated with an earlier and faster resurgence in weight gain velocity in early adulthood. Factors that contribute to an asynchronous timing of ages of peak weight and peak height velocities may accentuate the risk of adult obesity.

## Introduction

The prevalence of obesity is escalating globally, but more rapidly in low- and middle-income countries (LMIC) [1], which is contributing to an epidemiological transition of disease burden from communicable to non-communicable diseases [2, 3]. Obesity is associated with increased morbidity and mortality, partially through an increased risk of cardiometabolic diseases in adulthood. Increased waist circumference, body mass index (BMI) and whole-body fat mass are associated with markers of chronic inflammation [4] while increased truncal fat mass is associated with dyslipidaemia and insulin resistance [5]. When viewed through the life course, the prevalence of obesity rises in adolescence [6] and young adulthood [7], which are both critical periods for obesity. Thus, studying the factors that influence body composition during these critical periods may improve our understanding of the aetiology of obesity.

Physiological changes, which contribute to a central relocation of adipose tissue, and lifestyle changes during adolescence contribute to an obesogenic environment during this period [6]. Consequently, weight gain in childhood and adolescence may be more significant in influencing adult fat mass than weight gain in infancy [8], which is a critical period for the cardiometabolic disease risk [9]. During the childhood and adolescent period, data from the United Kingdom (UK) show that the incidence of obesity is highest in late childhood (7–11 years), while in South Africa it is highest in adolescence (11–18 years) [10]. The patterns of sex differences which emerge during adolescence may differ between LMIC and high-income countries (HIC) and these may influence the associations between adolescent and adult body composition. Sex differences in the prevalence of overweight and obesity vary from country to country with the United States of America (USA) having no sex differences [11], and Sweden a higher male prevalence [12], while our data from South Africa show that in females the prevalence rises with age while in males it remains constant through adolescence to early adulthood [13].

There’s paucity of data assessing the influence of the adolescent growth spurt for BMI on adult body composition in LMIC populations. Data from our group have shown that early puberty [14] and early BMI gain [15] are associated with higher BMI and risk of obesity in young adulthood. However, these studies did not assess the components of BMI (fat and lean mass) and did not characterise the adolescent growth spurt. It is suggested that the rates of adipose tissue accrual generally slow after peak height velocity [6], and few studies have used age at peak height velocity (APHV) as a marker in pubertal development to assess the period in adolescence at which adipose tissue accrual or weight gain may influence adult outcomes [7]. Therefore, the aims of this longitudinal study of South African urban black children through adolescence and into young adulthood were to assess the associations between the magnitude (size), timing (tempo) and intensity (velocity) of adolescent height, weight and BMI gain and adult fat mass, lean mass, height and weight.

## Materials and methods

### Study design and setting

Data from the prospective mixed-longitudinal Birth to Thirty (Bt30) birth cohort, which followed 3273 black and white children born between April and June 1990 in the Greater Johannesburg Municipality were used. Details of the cohort are described elsewhere but are briefly summarised here [16–18]. Mothers with a permanent residential address in the municipal region were asked to participate in the study and were recruited at different time points, initially at antenatal clinics, then at delivery, and at 3 months, 6 months, and one year post-delivery [16]. Data were collected at yearly data collection cycles from birth to 19 years. In young adulthood, data were collected between 21 and 24 years (21+ years) and a convenient sample of 1881 black participants who had data in young adulthood were included in the current study. All participants above the age of 18 years provided written informed consent while at younger ages, their guardians provided written informed consent and the participants provided assent (from 11 years). Ethics approval was obtained from the University of the Witwatersrand Committee for Research on Human Subjects (M010556).

### Anthropometric measurements

Height and weight were measured from 7 to 21+ years using the method of Lohman et al. [19]. Height was measured without shoes to the nearest 0.1 cm using a Harpenden Stadiometer (Holtain, U.K.). Weight was measured on an electronic scale to the nearest 0.1 kg. BMI was derived from height and weight. The International Obesity Task Force (IOTF) age and sex specific cut-offs for BMI were used to evaluate nutritional status (overweight/obese vs. normal/underweight) between the ages of 7 and 18 years [20]. The adult BMI cut-offs were used to determine nutritional status beyond 18 years.

### Body composition outcome variables

Whole body (excluding head because of hair weaves) composition was assessed by dual x-ray absorptiometry (DXA) using standard techniques. All scans were conducted by a qualified technician on a Hologic QDR 4500 A machine when participants were 21+ years old to estimate bone mineral content (BMC), fat-free mass (FFM), fat mass (FM), abdominal visceral adipose tissue (VAT), and abdominal subcutaneous adipose tissue (SAT). Fat-free soft tissue mass (FFSTM) was calculated as the difference between FFM and BMC, while the fat mass index (FMI) and fat-free soft tissue mass index (FFMI) were calculated by dividing FM (kg) and FFSTM (kg) by the square of height (m^2^), respectively. These scans were analysed using Hologic APEX 3.1 software [21]. A spine phantom was used for daily calibration, and coefficients of variation during the course of the study were less than 1% for total FM and FFSTM respectively. DXA-determined VAT and SAT were computed as described previously [21]. SAT was estimated by summing the subcutaneous fat measured from the DXA image on each side of the abdominal cavity, and subcutaneous fat overlying the abdominal cavity which was determined by modelling [21]. DXA-VAT was estimated by subtracting SAT from the total abdominal fat determined by DXA [21].

### Pubertal development assessment and trajectories

Pubertal development was assessed between the ages of 9 and 16 years using the Tanner sexual maturation scale (SMS). The scale assesses the development of secondary sexual characteristics (breasts in females, genitals in males, and pubic hair in both sexes) based on 5 progressive stages, from 1 (prepubertal) to 5 (post pubertal) depicted in photographs with written descriptions [22, 23]. A trained health care professional administered the assessment from ages 9–11 y, while participants performed a self-rated assessment from age 12 to 16 years. The determination of pubertal development was based on self-reported assessments using line drawings of the Tanner sexual maturation stages for pubic hair and breast/genital development as aids. Self-assessment has been validated in this setting [24]. Pubertal trajectory classes were computed from the Tanner sexual maturation scale data between ages 9 to 16 years using latent class growth analyses (LCGA). LCGA were conducted on girls and boys separately resulting in three classes for pubic hair development in each sex. For each sex, class 1 encompassed the group of children who had the lowest Tanner stage at 9 years of age and whose pubertal progression was slowest over time, class 3 included those with the earliest pubertal onset and fastest progression through puberty, and class 2 was defined as an intermediate group [25].

### SITAR modelling of adolescent growth

Height, weight and BMI from age 7 to 21+ years were modelled using the SuperImposition by Translation and Rotation (SITAR; Version 1.0.10 in R version 3.4.2), a shape invariant model with a single fitted curve that summarises individual growth patterns with three parameters [26]. The subject-specific random effects (α_i_, β_i_, γ_i_) correspond to the magnitude (size), timing (tempo) and intensity (velocity) of height, weight or BMI, and make individual curves as similar in shape as possible. The random effects allow for variation along the x and y axes in the units of the measurement, throughout the period of measurement i.e. 7 to 21+ years for the current study. Magnitude refers to a vertical shift on the y-axis which indicates how large or small an individual is relative to the sample mean curve. Timing refers to a horizontal shift on the x-axis, which indicates the onset of the different adolescent growth spurt variables. Age at peak height velocity was derived from the tempo (timing) random effect. Intensity gives an indication of slope of the curve to demonstrate how fast or slow the change is in an individual relative to the sample mean curve. Degrees of freedom for the best fitting model were chosen based on the lowest Bayesian Inclusion Criterion (BIC). The pubic hair classes were used to stratify participants according to the timing of development in SITAR models, presented as plots.

### Statistical analysis

Data were analysed using Stata version 13.1 (Stata-Corp LP, College Station, Texas, USA). Continuous variables were assessed for normality using the skewness and kurtosis test. The data is presented as means (SD) for continuous variables and frequencies (proportions) for categorical data. Sex differences in continuous variables were assessed using the independent t-test, with specification for unequal variances, while the Pearson chi-squared test was used to assess differences in categorical variables. The standard deviation test was used to assess equality of variances. To assess the association between the timing of weight or BMI gain and adult outcomes, the age at peak height velocity (APHV) was subtracted from the age at peak weight (APWV) or BMI (APBV) velocity. Using this difference, participants were grouped into tertiles for early, average and late peak weight or BMI velocity. One-way analyses of variance (ANOVA) with Bonferroni multiple comparisons test were used to assess differences in adult outcomes between the three groups. Multiple linear regression analyses were performed to assess adolescent factors associated with adult height, weight, BMI, FM, FFSTM, FMI, FFMI, VAT & SAT. Separate models for adolescent height, weight and BMI as exposures were performed. To adjust for pubertal development, the pubic hair development latent classes were included in the models. The variance inflation factor was used to assess multicollinearity. The Q-ladder function which performs a QQ-plot of residuals was used to assess appropriate transformation for dependent variables and all outcome variables were log transformed.

## Results

### Sex differences in body composition and size in adults

As adults (age 21–24 years old), males were taller and had greater FFSTM than females (*p* < 0.001). Females were slightly heavier (*p* < 0.05) and had greater BMI, FM, VAT and SAT (*p* < 0.001) (Table 1).

**Table 1:** Sex differences in adult body size and body composition.

Variables	Males	Females	*P* value^ϕ^
*N*	903	978	
Height (cm)	171.7^a^ (6.6)	159.7 (6.1)	<0.001
Weight (kg)	63.5 (11.3)	65.3 (16.2)	0.015
BMI (kg/m^2^)	21.5 (3.6)	25.6 (6.1)	<0.001
Fat mass (kg)	12.8 (6.1)	25.0 (9.9)	<0.001
Fat-free soft-tissue mass (kg)	44.1 (5.9)	33.7 (5.2)	<0.001
Fat mass-to-fat-free mass ratio	0.3 (0.1)	0.7 (0.2)	<0.001
Visceral adipose tissue (g)	222.0 (91.6)	288.9 (175.8)	<0.001
Subcutaneous adipose tissue (g)	451.7 (367.4)	1489.2 (718.8)	<0.001
Pubic hair development classes^b^			
Class 1 (slow developers)	188	178	
Class 2 (average developers)	394	405	
Class 3 (fast developers)	93	100	

^a^Mean ± SD; ^ϕ^Differences were assessed using a t-test with specification for unequal variances.

^b^Pubertal class 3 had the earliest onset and faster progression of pubic hair development while class 1 had the most delayed and slowest progression. Class 2 was intermediate.

### Effect of puberty (pubic hair development) on adolescent growth in height and weight and adult size (Fig. 1 and Supplementary Table 1)

During adolescence, males with the earliest or intermediate onset of pubic hair development (classes 2 and 3) were taller (*p* < 0.05) and heavier (*p* < 0.001) than those with a slowest onset (pubic hair class 1), but the differences in height disappeared in adulthood. In females, the timing of the onset of pubic hair development was not associated with early adult height. Both males and females with the earliest onset of pubic hair development (class 3) were heavier and had faster weight gain than those with later onset (*p* < 0.001). Females in the earliest onset group experienced a resurgence in weight gain in early adulthood. Early and average developers had an earlier timing of the growth spurts for height and weight in both sexes.

**Fig. 1:**
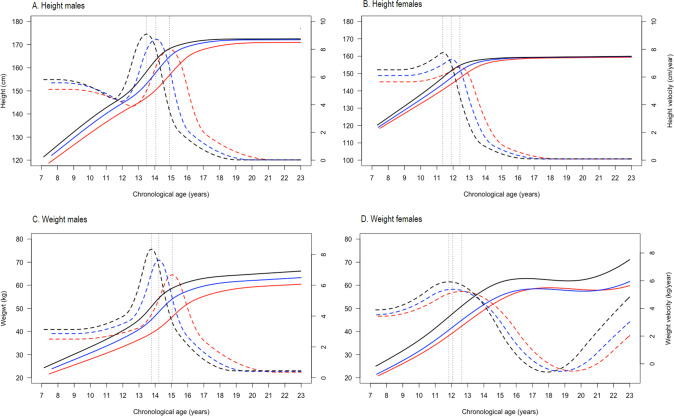
The effect of latent classes of pubic hair development on adolescent growth in height and weight. Height is shown in panels **A** and **B**, and weight in panels **C** and **D** for males and females, respectively. Pubic hair development was stratified into 3 classes: class 1 (red), class 2 (blue) and class 3 (black). Height males: APV and PV; class 1 (14.9 years and 8.9 cm/year), class 2 (14.1 years and 8.7 cm/year) and class 3 (13.5 years and 9.1 cm/year); height females: APV and PV; class 1 (12.4 years and 6.7 cm/year), class 2 (11.9 years and 7.3 cm/year) and class 3 (11.4 years and 7.7 cm/year); weight males: APV and PV; class 1 (15.1 years and 6.7 kg/year), class 2 (14.2 years and 7.7 kg/year) and class 3 (13.8 years and 8.4 kg/year); and weight females: APV and PV; class 1 (12.6 years and 5.2 kg/year), class 2 (12.1 years and 5.4 kg/year) and class 3 (11.8 years and 5.9 kg/year).

### Adolescent height growth and adult body composition and size (Table 2a)

The magnitude of adolescent height gain was positively associated with adult height, weight, FM, and FFSTM in both sexes, as well as FMI, VAT and SAT in males. In both sexes, the later onset (timing) of adolescent height gain was associated with a reduction in all measures of adult body composition and weight, but not height in males. However, in females an earlier timing of adolescent height gain was associated with taller adult height. Faster height gain was associated with taller adult height and VAT/SAT ratio, but lower weight and all measures of body composition in males. The intensity of height gain in females had no influence on final body composition or size. Those with the earliest onset of pubic hair development (class 3) had greater adult weight, BMI, fat mass and FMI than later developers. (Table 2). Although alone pubic hair development (Supplementary Table 1) had no effect on height in females, but when assessed with the magnitude, timing and intensity of adolescent height gain, the earliest onset of pubic hair development was associated with shorter adult height (Table 2a).

**Table 2:** Linear regression analyses to assess the effect of the magnitude, timing and intensity of adolescent growth in (a) height, (b) weight, and (c) BMI on adult outcomes, adjusted for pubic hair development classes.

(a) Adolescent height										
Adolescent variables	Adult variables									
	Height (cm)	Weight (kg)	BMI (kg/m^2^)	Fat mass (g)	Fat free mass (g)	FMI (kg/m^2^)	FFMI (kg/m^2^)	VAT (g)	SAT (g)	VAT/SAT Ratio
Males										
Magnitude	1.005***	0.013***	0.001	0.019***	0.013***	0.007*	0.001	0.008*	0.017**	−0.009*
	(0.009)	(0.001)	(0.001)	(0.003)	(0.001)	(0.003)	(0.001)	(0.004)	(0.006)	(0.004)
Timing	–0.095	–0.057***	–0.056***	–0.129***	–0.042***	–0.129***	–0.041***	–0.107***	−0.194***	0.089*
	(0.073)	(0.009)	(0.009)	(0.029)	(0.008)	(0.029)	(0.008)	(0.030)	(0.048)	(0.036)
Intensity	2.800***	–0.353***	–0.384***	–0.954***	–0.171*	–0.985***	–0.204**	–0.647**	–1.364***	0.743*
	(0.597)	(0.071)	(0.071)	(0.238)	(0.067)	(0.238)	(0.067)	(0.249)	(0.396)	(0.299)
Pubic hair 2	–0.011	0.016	0.016	0.060	–0.021	0.060	–0.021	0.081	0.051	0.030
	(0.118)	(0.014)	(0.014)	(0.047)	(0.013)	(0.047)	(0.013)	(0.049)	(0.078)	(0.058)
Pubic hair 3	–0.273	0.053**	0.056**	0.130	–0.002	0.134*	0.002	0.052	0.155	–0.104
	(0.172)	(0.020)	(0.020)	(0.067)	(0.019)	(0.067)	(0.019)	(0.070)	(0.112)	(0.084)
R-squared	0.967	0.235	0.094	0.098	0.324	0.072	0.069	0.050	0.058	0.030
Females										
Magnitude	1.009***	0.013***	0.001	0.011**	0.013***	−0.002	0.001	0.008	0.004	0.003
	(0.009)	(0.002)	(0.002)	(0.003)	(0.001)	(0.003)	(0.001)	(0.006)	(0.004)	(0.003)
Timing	–0.222**	–0.082***	–0.079***	−0.137***	–0.037***	–0.135***	–0.035***	–0.212***	–0.179***	–0.033
	(0.085)	(0.015)	(0.015)	(0.031)	(0.010)	(0.031)	(0.010)	(0.053)	(0.040)	(0.030)
Intensity	0.886	–0.141	–0.152	−0.271	–0.080	–0.284	–0.094	−0.418	−0.295	–0.122
	(0.505)	(0.087)	(0.087)	(0.194)	(0.065)	(0.195)	(0.066)	(0.333)	(0.253)	(0.188)
Pubic hair 2	–0.099	–0.023	–0.022	−0.041	0.001	–0.041	0.001	−0.115	−0.062	–0.053
	(0.117)	(0.020)	(0.020)	(0.044)	(0.014)	(0.044)	(0.015)	(0.073)	(0.055)	(0.041)
Pubic hair 3	–0.463**	0.047	0.053	0.041	0.019	0.047	0.025	−0.053	0.035	–0.087
	(0.167)	(0.029)	(0.029)	(0.061)	(0.020)	(0.061)	(0.020)	(0.101)	(0.077)	(0.057)
R-squared	0.958	0.136	0.083	0.071	0.263	0.084	0.041	0.052	0.076	0.008

(b) Adolescent weight										
Males										
Magnitude	26.450***	1.065***	0.757***	2.016***	0.811***	1.701***	0.501***	1.504***	2.701***	–1.200***
	(1.893)	(0.024)	(0.033)	(0.121)	(0.029)	(0.129)	(0.032)	(0.142)	(0.219)	(0.187)
Timing	–1.277***	–0.003	0.012*	–0.039*	0.005	–0.027	0.017***	–0.022	–0.045	0.024
	(0.292)	(0.004)	(0.005)	(0.018)	(0.004)	(0.020)	(0.005)	(0.022)	(0.033)	(0.028)
Intensity	–13.109***	0.038	0.190***	–0.048	0.037	0.096	0.182***	0.079	0.109	–0.023
	(2.273)	(0.029)	(0.039)	(0.142)	(0.034)	(0.152)	(0.038)	(0.169)	(0.258)	(0.221)
Pubic hair 2	0.656	–0.000	–0.008	0.018	–0.014	0.009	–0.023*	0.051	−0.019	0.071
	(0.546)	(0.007)	(0.009)	(0.034)	(0.008)	(0.037)	(0.009)	(0.041)	(0.062)	(0.053)
Pubic hair 3	–0.488	0.023*	0.029*	0.044	–0.005	0.050	–0.000	0.003	0.043	–0.040
	(0.793)	(0.010)	(0.014)	(0.049)	(0.012)	(0.053)	(0.013)	(0.058)	(0.090)	(0.076)
R-squared	0.233	0.797	0.570	0.477	0.715	0.381	0.509	0.286	0.346	0.129
Females										
Magnitude	13.117***	1.080***	0.916***	1.718***	0.717***	1.543***	0.537***	2.205***	1.836***	0.369**
	(1.719)	(0.033)	(0.039)	(0.092)	(0.032)	(0.101)	(0.032)	(0.195)	(0.132)	(0.132)
Timing	−0.532	–0.005	0.002	−0.008	–0.007	0.006	0.006	–0.017	0.010	–0.027
	(0.301)	(0.006)	(0.007)	(0.015)	(0.005)	(0.017)	(0.005)	(0.033)	(0.022)	(0.023)
Intensity	–7.187**	0.118**	0.208***	0.279*	–0.023	0.404**	0.103*	0.294	0.582***	–0.288
	(2.289)	(0.043)	(0.052)	(0.119)	(0.043)	(0.131)	(0.042)	(0.258)	(0.174)	(0.174)
Pubic hair 2	0.414	0.012	0.007	0.010	0.021*	0.002	0.015	−0.046	−0.007	–0.040
	(0.537)	(0.010)	(0.012)	(0.028)	(0.010)	(0.031)	(0.010)	(0.060)	(0.040)	(0.040)
Pubic hair 3	–0.095	0.013	0.014	–0.012	0.003	−0.008	0.008	–0.075	0.011	–0.086
	(0.759)	(0.014)	(0.017)	(0.039)	(0.014)	(0.043)	(0.014)	(0.082)	(0.055)	(0.055)
R-squared	0.093	0.773	0.661	0.602	0.632	0.526	0.554	0.352	0.499	0.023

(c) Adolescent BMI										
Males										
Magnitude	–0.982	1.021***	1.033***	2.477***	0.598***	2.473***	0.596***	1.861***	3.611***	–1.756***
	(3.164)	(0.052)	(0.037)	(0.176)	(0.062)	(0.170)	(0.044)	(0.204)	(0.304)	(0.267)
Timing	–0.176	–0.001	0.001	–0.018*	0.003	−0.016*	0.005**	−0.013	−0.023	0.010
	(0.142)	(0.002)	(0.002)	(0.008)	(0.003)	(0.008)	(0.002)	(0.010)	(0.014)	(0.013)
Intensity	–1.637	–0.037*	−0.018	–0.092	0.006	−0.085	0.013	−0.004	−0.128	0.126
	(1.092)	(0.018)	(0.013)	(0.059)	(0.021)	(0.056)	(0.015)	(0.068)	(0.101)	(0.089)
Pubic hair 2	1.576**	0.019	0.000	0.053	–0.003	0.037	−0.020*	0.076*	0.031	0.046
	(0.591)	(0.010)	(0.007)	(0.033)	(0.012)	(0.031)	(0.008)	(0.038)	(0.057)	(0.050)
Pubic hair 3	1.109	0.033*	0.020*	0.063	0.005	0.054	−0.006	0.014	0.066	–0.052
R-squared	0.024	0.576	0.746	0.489	0.382	0.512	0.581	0.316	0.408	0.165
Females										
Magnitude	–4.137*	0.956***	1.008***	1.580***	0.504***	1.655***	0.571***	2.082***	1.887***	0.195
	(1.828)	(0.042)	(0.035)	(0.107)	(0.048)	(0.101)	(0.034)	(0.217)	(0.144)	(0.150)
Timing	0.256	–0.009**	−0.012***	–0.014	–0.002	−0.015	−0.003	−0.022	−0.020	−0.002
	(0.148)	(0.003)	(0.003)	(0.008)	(0.004)	(0.008)	(0.003)	(0.017)	(0.011)	(0.011)
Intensity	0.167	0.073**	0.070***	0.144**	0.020	0.163**	0.033	0.215	0.200**	0.015
	(0.984)	(0.023)	(0.019)	(0.055)	(0.025)	(0.052)	(0.018)	(0.111)	(0.074)	(0.076)
Pubic hair 2	0.309	0.011	0.007	0.017	0.020	0.012	0.015	–0.035	0.009	–0.044
	(0.548)	(0.013)	(0.010)	(0.029)	(0.013)	(0.028)	(0.009)	(0.058)	(0.038)	(0.040)
Pubic hair 3	1.129	0.029	0.015	0.012	0.019	–0.002	0.007	–0.056	0.027	–0.084
	(0.775)	(0.018)	(0.015)	(0.040)	(0.018)	(0.038)	(0.013)	(0.079)	(0.053)	(0.055)
R-squared	0.020	0.633	0.738	0.561	0.341	0.616	0.579	0.371	0.528	0.015

Data presented as coefficients (SE). Significance is indicated by asterisks.

Standard errors in parentheses

***p < 0.001, **p < 0.01, *p < 0.05

The variance explained by the model (magnitude, timing and intensity of adolescent height and pubic hair development classes) was approximately 96% for adult height in both sexes, 23.5% for adult weight in males and 13.6% in females and 32.4% for FFSTM in males and 26.3% in females. Adolescent height gain explained less than 10% of the variance in the other adult variables.

### Adolescent weight gain and adult body composition and size (Table 2b)

The magnitude of adolescent weight gain was positively associated with adult height and all measures of adult body composition in both sexes.

In males, the timing of adolescent weight gain was negatively associated with adult height and fat mass but positively associated BMI and the FFMI. In males and females, the intensity of adolescent weight gain was negatively associated with adult height, but positively with a number of variables associated with body composition, such as BMI and fat free soft tissue mass (Table 2b).

The variance in adult height explained by the model (magnitude, timing and intensity of adolescent weight and pubic hair development classes) was ~23% and 9% in males and females respectively. Between ~29% and ~72% and between ~35% and ~ 66% of the variance in adult body composition (excluding VAT/SAT ratio) could be explained by the parameters of adolescent weight gain and pubic hair development classes in males and females, respectively.

### Adolescent BMI gain and adult body composition and size (Table 2c)

The magnitude of adolescent BMI gain has the greatest associations with adult body composition variables in both males and females. The other measures of adolescent BMI gain (timing and intensity) had few significant associations with adult height or body composition, except for adult weight and BMI in females. An earlier timing of adolescent BMI gain in males was associated with a significant increase in FMI but a reduction in FFMI.

Between ~32% and ~75% and between ~37% and ~74% of the variance in adult body composition (excluding VAT/SAT ratio) could be explained by the parameters of adolescent BMI gain and pubic hair development classes, in males and females respectively.

### Effects of the timing of peak weight velocity and peak BMI velocity in relation to age at peak height velocity on adult outcomes (Table 3)

An earlier peak of weight velocity relative to APHV was associated with taller adult height but lower weight, BMI and FFMI (Table 3a). In males, an early peak weight velocity relative to APHV was associated with lower FFSTM while in females it was associated with lower FM. In females, early peak weight velocity relative to APHV was associated with lower FMI, FFMI, VAT and SAT.

**Table 3:** The timing of peak velocity for (a) weight gain, and (b) BMI gain in relation to age at peak height velocity and their effects on adult body composition and height.

(a) Adolescent weight gain						
Variables	Males			Females		
	Early	Intermediate	Late	Early	Intermediate	Late
Range of difference^ϕ^ (years)	–0.52 (–8.51 to –0.11) [340]	0.14 –0.11 to 0.38 [339]	0.66 0.38–4.70 [339]	–0.02 –3.09 to 0.36 [363]	0.68 0.36–0.98 [363]	1.42 0.98–4.33 [363]
Age at peak height velocity (years)	14.5^a**, b***^ (1.2) [310]	14.2^c***^ (1.0) [313]	13.9 (0.9) [280]	11.9 ^a**,b***^ (1.0) [312]	11.8^c***^ (0.8) [325]	11.5 (0.8) [341]
Age at peak weight velocity (years)	13.6^a***, b***^ (1.3) [310]	14.3^c**^ (0.9) [313]	14.6 (1.0) [280]	11.8^a***, b***^ (1.0) [312]	12.4^c***^ (0.8) [325]	13.0 (1.0) [341]
Peak weight velocity (kg/year)	6.9^a***, b**^ (0.2) [310]	6.8 (0.1) [313]	6.8 (0.1) [280]	6.4^b**^ (0.2) [312]	6.3 (0.1) [325]	6.3 (0.1) [341]
Overweight/obesity^!^ (%)	107 (34.5) [310]	109 (34.8) [313]	108 (38.6) [280]	155 ^a**, b***^ (49.7) [312]	206^c**^ (53.4) [324]	251 (73.6) [340]
Adult Height (cm)	172.8^b***^ (6.9) [233]	171.6 (6.4) [223]	170.4 (6.4) [224]	161.3^b***^ (6.0) [223]	160.0^c**^ (5.8) [215]	158.3 (6.2) [257]
Adult Weight (kg)	62.9^b**^ (11.4) [233]	60.9^c***^ (9.5) [222]	66.7 (12.1) [223]	61.9^b***^ (17.0) [220]	63.8^c***^ (13.8) [214]	69.7 (16.5) [255]
Adult BMI (kg/m^2^)	21.1^b***^ (3.7) [233]	20.6^c***^ (2.7) [222]	22.9 (3.9) [223]	23.8^b***^ (6.4) [220]	24.9^c***^ (5.2) [214]	27.8 (6.0) [255]
Fat mass (g)	13040.0 (6846.9) [150]	11765.2^c***^ (5388.4) [161]	13993.4 (6462.8) [147]	22272.5^b***^ (10127.7) [146]	24055.4^c***^ (9101.5) [157]	28481.1 (9546.9) [163]
Fat-free soft tissue mass (g)	43091.7^b***^ (5577.0) [158]	43592.2^c**^ (5768.0) [164]	45831.3 (6206.5) [154]	33128.4 (5424.1) [154]	33512.1 (4865.9) [165]	34240.3 (5058.7) [168]
FMI (kg/m^2^)	4.4 (2.3) [146]	3.9^c***^ (1.6) [157]	4.8 (2.1) [139]	8.5^b***^ (3.7) [140]	9.4^c***^ (3.6) [150]	11.5 (3.8) [159]
FFMI (kg/m^2^)	14.5^b***^ (1.6) [154]	14.7^c***^ (1.5) [160]	15.8 (1.8) [146]	12.6^a*, b***^ (1.7) [147]	13.2^c**^ (1.7) [158]	13.8 (1.7) [164]
Fat mass-to-fat free mass ratio	0.30^a*^ (0.14) [150]	0.27^c*^ (0.10) [161]	0.30 (0.11) [147]	0.66^b***^ (0.22) [146]	0.71 (0.21) [157]	0.83 (0.22) [163]
Visceral adipose tissue mass (g)	468.3 (436.9) [156]	383.4 (311.8) [161]	518.5 (393.6) [148]	1253.2^b***^ (751.4) [146]	1408.7^c***^ (677.9) [156]	1722.3 (691.8) [163]
Subcutaneous adipose tissue mass (g)	217.6 (98.3) [156]	209.1^c**^ (92.6) [161]	234.6 (96.4) [148]	239.9^b***^ (168.7) [146]	276.2^c***^ (177.3) [156]	336.4 (174.8) [163]

(b) Adolescent BMI gain						
Range of difference^ϕ^ (years)	–2.54 –9.92 to –1.54 [340]	–0.79 –1.51 to 0.08 [339]	1.11 0.08–8.45 [339]	0.53 –3.17 to 1.35 [363]	1.83 1.35–2.35 [363]	3.09 2.35–8.11 [363]
Age peak of height velocity (years)	14.6^a***, b***^ (1.2) [328]	14.1^c***^ (0.9) [299]	13.7 (0.9) [276]	11.9^b***^ (1.0) [324]	11.7^c*^ (0.8) [327]	11.6 (0.8) [327]
Age peak of BMI velocity (years)	11.7^a***, b***^ (1.3) [328]	13.3^c***^ (1.0) [299]	15.4 (1.7) [276]	12.2^a***, b***^ (1.3) [324]	13.6^c***^ (0.8) [327]	15.0 (1.3) [327]
Peak BMI velocity (kg/m^2^/year)	0.8^a*, b***^ (0.3) [328]	0.9^c***^ (0.3) [299]	1.1 (0.3) [276]	1.4^a***, b***^ (0.3) [324]	1.2^c***^ (0.3) [327]	1.1 (0.3) [327]
Overweight/obesity^!^ (%)	83^a***, b***^ (25.3) [328]	122 (40.8) [299]	119 (43.1) [276]	232^b***^ (71.6) [324]	215^c***^ (65.8) [327]	165 (50.5) [327]
Height (cm)	172.4^b*^ (6.9) [260]	171.8 (6.2) [196]	170.7 (6.5) [224]	160.5 (6.3) [231]	159.4 (6.1) [215]	159.4 (5.9) [249]
Weight (kg)	59.6^a*, b***^ (8.9) [260]	62.4^c***^ (10.4) [196]	69.1 (12.3) [222]	70.7^a***, b***^ (18.3) [227]	64.0 (14.9) [213]	61.7 (13.9) [249]
BMI (kg/m^2^)	20.0^a**, b***^ (2.7) [260]	21.1^c***^ (3.2) [196]	23.7 (3.8) [222]	27.4^a***, b***^ (6.8) [227]	25.2 (5.9) [213]	24.3 (5.2) [249]
Fat mass (g)	11184.8^b***^ (4963.9) [168]	12128.5^c***^ (5968.7) [138]	15518.3 (7061.7) [151]	27386.9^a*, b**^ (11211.1) [149]	24554.8 (9404.1) [147]	23415.9 (8751.6) [170]
Fat-free soft tissue mass (g)	41506.3^a***, b***^ (4805.3) [177]	44165.1^c***^ (5428.5) [141]	47099.5 (6213.7) [158]	34955.9^a**, b***^ (5425.9) [160]	33166.2 (4678.3) [151]	32855.7 (5001.8) [176]
FMI (kg/m^2^)	3.8^b***^ (1.7) [166]	4.1^c***^ (2.0) [132]	5.2 (2.2) [144]	10.7^b**^ (4.4) [143]	9.8(3.8) [140]	9.2 (3.5) [166]
FFMI (kg/m^2^)	14.0^a***, b***^ (1.3) [174]	15.0^b***^ (1.4) [135]	16.1 (1.7) [151]	13.6^b**^(1.9) [153]	13.2(1.6) [144]	12.9(1.8) [172]
Fat mass-to-fat free mass ratio	0.27^b***^ (0.10) [169]	0.27^c***^ (0.12) [139]	0.33 (0.13) [151]	0.77^b*^ (0.25) [149]	0.73 (0.22) [147]	0.71 (0.21) [170]
Visceral adipose tissue mass (g)	364.4^b***^ (304.6) [173]	414.4^c***^ (376.5) [139]	593.9 (438.8) [153]	1607.0 (846.0) [155]	1455.1 (644.1) [141]	1356.2 (668.3) [169]
Subcutaneous adipose tissue mass (g)	194.1^b***^ (75.4) [173]	218.1^c***^ (98.4) [139]	251.3 (106.0) [153]	311.5^b**^ (195.9) [155]	281.6 (164.3) [141]	265.9 (169.5) [169]

Results are presented as mean (SD), unless otherwise stated. Sample size is in square brackets.

^ϕ^The difference was calculated by subtracting age at peak height velocity from age at peak weight or BMI velocity (e.g. apwv—aphv). This value was categorised by generating tertiles for early, intermediate and late age at peak weight or BMI velocity. Data presented as median (range).

^!^Data presented as count (percentage).

^a^Early vs. intermediate.

^b^Early vs. late.

^c^Intermediate vs. late.

*p < 0.05, **p < 0.01, ***p < 0.001.

The effect of timing of peak BMI velocity in relation to peak height velocity on adult anthropometry was diametrically opposite in the two sexes. Early onset peak BMI velocity resulted in decreased adult FM, FFSTM, FMI, FFMI, VAT and SAT in males, but the converse was found in females (Table 3b). In males, weight, BMI, FM and FMI were lowest for those who had their BMI peak around APHV.

## Discussion

We investigated the association between pubertal development and adolescent growth and their association with young adult height and body composition. While adolescent body composition may reflect concurrent metabolic changes in puberty [27], both the timing and magnitude of adolescent BMI gain are associated with adult morbidity. Dietz suggested that the association between adolescent obesity and morbidity may be driven by an increase in total body fat and redistribution of fat mass to central depots during puberty [28]. We measured whole body and abdominal (VAT & SAT) fat mass, whole body fat-free mass, weight and BMI in early adulthood and found that the magnitude of weight and BMI gain achieved in adolescence was better at predicting adult body composition than intensity and timing. These results support He and Karlberg’s findings that BMI status, i.e. BMI achieved at a particular age, was more important for subsequent body size than the rate of BMI gain [29]. However, the intensity of adolescent weight and BMI gain were also positively associated with adult weight, BMI, fat mass, FMI and SAT but not VAT, although not to the same degree as the magnitude of weight and BMI gain. The association of the timing of adolescent height gain, where early height gain was associated with greater whole-body fat and fat-free mass and both abdominal SAT and VAT, is supported by previous findings [30].

Adolescence is a critical period for obesity, which may predispose to increased risk of excess adiposity in this period and beyond. Thus, assessing changes in adolescent BMI may give insight into drivers of excess fat. BMI is easy to measure, making it a useful but crude proxy for adiposity. It has been reported that FMI and FFMI have no additional value in predicting the metabolic syndrome over BMI in children and adolescents [31]. However, the sensitivity of overweight, as determined by BMI, in measuring excess FMI is influenced by sex and ethnicity. Weber et al. found that the positive predictive value of overweight status in measuring excess FMI was considerably lower in black (35.9% & 30.3%) than white (65.4% & 52.2%) and Mexican American (73.3% & 68.3%) adolescent males and females, respectively [32]. There are also ethnic differences in fat distribution with African American children having lower visceral fat but similar subcutaneous and whole-body fat than white American children [33]. We were unable to confirm this, due to the low number of white participants in the 21 to 24-year age group, who were thus not included in the current study. Notwithstanding, the nutrition transition could be more prominent in the black population in South Africa, with black females having an increasingly higher prevalence of overweight and obesity [13] and BMI velocity [34] in adolescence than South African white females.

The current study adds to our knowledge of the contribution of adolescence to the risk of both excess fat mass (whole body and abdominal) and BMI gain in adulthood. There is a growing interest in the age group 18 to 25 years [35] as these early adult years may represent another critical period for the rise in obesity. Our previous findings on this cohort show that black females experience a resurgence in BMI gain in early adulthood period (18+ years), which is faster than that in white females [34]. In the current study, a similar resurgence in weight velocity in females was observed among those with early onset of pubic hair development when the data were stratified by pubertal development. These women were heavier in childhood through adolescence and had the earliest onset of puberty and age at peak weight velocity. Thus, a cascade of events starting with increased childhood adiposity to the earliest onset of puberty, and greater and earlier weight gain in adolescence leads to a resurgence of weight gain in the early adulthood period. Differences in BMI between those who become overweight before or after the age of 25 years emerge in mid-childhood [36] underscoring the persistence of early onset obesity through adolescence to adulthood.

The importance of childhood weight gain in females is emphasised in the sex differences in the association of the timing of peak BMI gain relative to APHV with adult outcomes. The age at peak BMI velocity (APBV) is an important landmark in the development of adolescent adiposity as Guo and colleagues found that BMI size at maximum velocity was the most important predictor of adult total body fat and percent body fat [37]. It has also been hypothesised that the rate of adiposity gain decreases after APHV [6]. Thus, assessing these patterns in relation to biological maturity aligns individuals on a common landmark to adjust for variations in developmental trajectories in order to estimate true phenotypic differences and their potential impact. Sex differences in adolescent height, weight and BMI were accentuated when observed against biological maturity than against chronological age [38]. When combined in a model with maturational status, chronological age was not significantly associated with weight status while early maturity was associated with greater weight status [39], emphasising the importance of maturational status in assessing these patterns. In this study, we found that APBV earlier than APHV was associated with greater early adult BMI, whole-body fat mass, FMI and subcutaneous adipose tissue in females but the reverse was true in males. Guo et al. found that BMI patterns during and post adolescence were more important for adult total body fat and percent body fat status than earlier patterns for both sexes while for women, childhood patterns were also important [37]. Thus, an asynchrony of weight, body composition and height gain in adolescence may predispose to increased risk of adult obesity.

The timing of peak velocity for weight (APWV) and components of body composition (fat mass and fat-free mass) are said to occur around APHV [40], although others have observed delays in attainment of APWV relative to APHV [41]. Previously in this cohort, we found delays of between 0.9 and 1.7 years in the timing of APWV & APBV compared to APHV [34]. Findings from the current show that a synchronised APHV and APWV contributed to the lowest BMI and fat mass in both sexes. In males, we also found that reaching APBV around APHV was associated with the lowest early adult weight, BMI, FM and FMI. This supports findings by Barbour-Tuck et al., who showed that overweight status at APHV was not associated with early adult overweight status in a cohort of adolescent males [7]. In females, the earliest APBV group, who had the highest BMI and fat mass, paradoxically had a later APHV. This is surprising given that a greater childhood or early adolescent BMI is associated with an earlier APHV [42, 43]. Understanding factors that contribute to an asynchronous timing of the adolescent growth spurts for height, weight and BMI may improve our understanding of the lifecourse determinants of obesity.

There are a number of strengths of this paper. Firstly, we used multiple indicators of adolescent growth and pubertal development to assess their associations and how they relate to adult outcomes. The SITAR parameters of size (magnitude), tempo (timing) and velocity (intensity) which provide a multidimensional assessment of adolescent growth add to the strengths of this study. In addition, we used pubic hair development to provide an independent measure of the timing of pubertal development. Another strength of this paper is that the data are from a middle-income country where there is a rapid rise in obesity. We also used several indicators of body composition outcome including BMI and DXA derived body composition, which are scarce in these settings. However, the study does have limitations. The sample was exclusively made up of black urban South Africans and thus the findings may not be generalisable to other populations or rural communities. In addition, we did not adjust our analyses for lifestyle factors such as socio-economic status, diet or physical activity. The low number of participants who developed overweight/obesity at APHV may influence the strength of the findings.

In conclusion, we found that the timing of the adolescent growth spurt for height was negatively associated with early adult BMI, whole body and abdominal fat mass. The magnitude of adolescent weight and BMI gain was more important in determining early adult body composition than the rate and timing of gain. However, early weight gain, before APHV, was an important determinant of increased BMI, and whole body and abdominal fat mass. Factors that contribute to an asynchronous timing of the adolescent growth spurts for height, weight and BMI may contribute to an increased risk of obesity, especially in females. Young adult females also experienced a resurgence in weight gain in early adulthood which was earlier and faster among those who were heavier in childhood and adolescence. The period of transition between the teenage years and early adulthood may be a critical period in which intervention may influence the risk of obesity and disease in later life. Strategies to limit the rate of weight gain during late childhood and adolescence might be beneficial for future metabolic health.

## Supplementary information

Supplementary Table 1

## Data Availability

The deidentified data that were used for generating the results in this manuscript can be made available upon request.
