# Supplementary material for: The association between the timing, intensity and magnitude of adolescent growth and body composition in early adulthood
Source: Eur J Clin Nutr. 2023 Jun 13;78(11):995–1004. doi: 10.1038/s41430-023-01293-9 (PMC11537958; doi:10.1038/s41430-023-01293-9)
Supplement: Supplementary file 1 — Supplementary Table 1 [file 41430_2023_1293_MOESM1_ESM.docx]

**Supplementary tables**

**Supplementary Table 1: Differences in the size (magnitude), tempo (timing) and velocity (intensity) of adolescent changes in height, weight and BMI between groups of** **pubertal development latent classes; class 1 (late), class 2 (average) and class 3 (early). The later development group (class 1) is the reference.**

| **Variable** | **SITAR Parameter** | **Males** | | **Females** | |
| --- | --- | --- | --- | --- | --- |
|  |  | **Early** | **Average** | **Early** | **Average** |
| N: participants/observations | | 921/6647 | | 859/6380 | |
| Height (cm) | Size | 1.50 (0.74)* | 1.13 (0.52)* | 0.29 (0.64) | 0.24 (0.48) |
|  | Tempo | -1.42 (0.11)*** | -0.8 (0.08)*** | -1.23 (0.11)*** | -0.63 (0.08)*** |
|  | Velocity | 0.13 (0.02)*** | 0.09 (0.02)*** | 0.14 (0.02)*** | 0.08 (0.01)*** |
| Weight (kg) | Size | 4.37 (1.02)*** | 2.07 (0.71)** | 4.00 (1.22)*** | -0.46 (0.91) |
|  | Tempo | -1.29 (0.16)*** | -0.84 (0.11)*** | -0.92 (0.17)*** | -0.60 (0.13)*** |
|  | Velocity | 0.22 (0.04)*** | 0.13 (0.03)*** | 0.12 (0.03)*** | 0.03 (0.02) |
| BMI (kg/m^2) | Size | 1.19 (0.26)*** | 0.30 (0.18) | 1.67 (0.42)*** | -0.15 (0.31) |
|  | Tempo | -0.57 (0.29)* | -0.57 (0.20)** | -1.00 (0.19)*** | -0.47 (0.15)** |
|  | Velocity | 0.29 (0.08)*** | 0.12 (0.06)* | 0.12 (0.06)* | -0.004 (0.04) |

*p < 0.05, **p < 0.01, ***p < 0.001
